# Estimating averted COVID-19 cases, hospitalisations, intensive care unit admissions and deaths by COVID-19 vaccination, Italy, January−September 2021

**DOI:** 10.2807/1560-7917.ES.2021.26.47.2101001

**Published:** 2021-11-25

**Authors:** Chiara Sacco, Alberto Mateo-Urdiales, Daniele Petrone, Matteo Spuri, Massimo Fabiani, Maria Fenicia Vescio, Marco Bressi, Flavia Riccardo, Martina Del Manso, Antonino Bella, Patrizio Pezzotti, on behalf of the Italian Integrated Surveillance of COVID-19 study group

**Affiliations:** 1 Department of Infectious Diseases, Istituto Superiore di Sanità, Rome, Italy; 2 The members of the Italian Integrated Surveillance of COVID-19 study group are acknowledged at the end of the article

**Keywords:** SARS-CoV-2, vaccines, impact, averted events, Italy

## Abstract

We assessed the impact of COVID-19 vaccination in Italy, by estimating numbers of averted COVID-19 cases, hospitalisations, ICU admissions and deaths between January and September 2021, by age group and geographical macro areas. Timing and speed of vaccination programme implementation varied slightly between geographical areas, particularly for older adults. We estimated that 445,193 (17% of expected; range: 331,059−616,054) cases, 79,152 (32%; range: 53,209−148,756) hospitalisations, 9,839 ICU admissions (29%; range: 6,434−16,276) and 22,067 (38%; range: 13,571−48,026) deaths were prevented by vaccination.

The roll-out of the vaccination against coronavirus disease (COVID-19) was launched in Italy on 27 December 2020 [1] and by the end of September 2021, 84% of the eligible population aged 12 years and older, had received at least one dose of a vaccine against COVID-19. National [2,3] and international [4] researchers have found high levels of vaccine effectiveness (VE) against severe acute respiratory syndrome coronavirus (SARS-CoV-2) infection and severe COVID-19. 

We evaluated the direct impact of the Italian vaccination programme on the number of cases, on hospitalisations, on admissions to intensive care units (ICU) and on deaths, by estimating the numbers of these outcomes prevented (averted events) by COVID-19 vaccination between January (week 2/2021) and the end of September 2021 (week 38/2021) by age groups and geographical macro area.

## Vaccine deployment and uptake 

The target groups for COVID-19 vaccination followed the recommendations of the Ministry of Health [5], with healthcare workers, residents in long-term care facilities and persons aged over 80 years being the first to receive the vaccines. Successively, the vaccine roll-out was extended to clinically extremely vulnerable groups and younger age groups in descending order, prioritising those with multiple comorbidities. The present vaccination programme in Italy targets the whole population aged 12 years and older with access to the national healthcare. About 80% of the vaccinated population has received the mRNA vaccines Cominarty (BNT162b2 mRNA, BioNTech-Pfizer, Mainz, Germany/New York, United States (US)) or Spikevax (mRNA-1273, Moderna, Cambridge, US), whereas the rest of the population has received Vaxzevria (ChAdOx1 nCoV-19, Oxford-AstraZeneca, Cambridge, United Kingdom (UK) or COVID-19 Vaccine Janssen (Ad26.COV2-S, Janssen-Cilag International NV, Beerse, Belgium).

**Figure 1:**
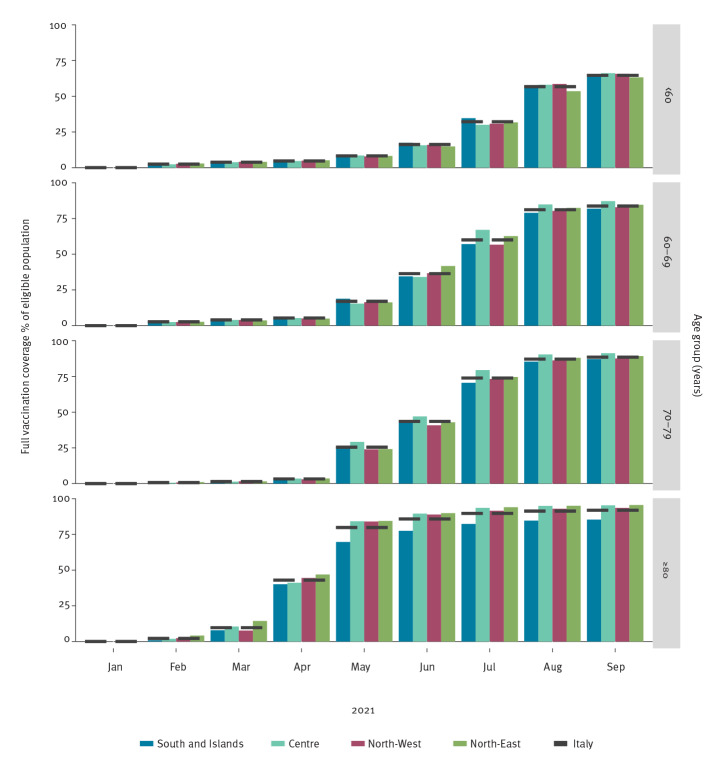
Cumulative monthly full vaccination coverage by age group and geographical macro area^a^, Italy, week 2/2021−week 38/2021

There was notable heterogeneity in the pace of vaccine uptake both across Italian regions and across Italian macro areas (North-West, North-East, Centre, and South-Islands, based on nomenclature of territorial units for statistics (NUTS1) areas for Italy [6]). While vaccine uptake was faster in the Centre of Italy, particularly in those aged 60–79 years, the South-Islands area has consistently reached lower levels of vaccine uptake in those aged 80 years and older compared the other macro areas (Figure 1). By the end of September (week 38), 65% (ranging from 63% in the North-East to 66% in the North-West and in the Centre) of those aged under 60 years, 84% (ranging from 82% in the South-Islands to 87% in the Centre) of those aged between 60 and 69 years, 89% (ranging from 87% in the South-Islands to 91% in the Centre) of those aged between 70 and 79 years and 92% (ranging from 85% in the South-Islands to and 96% in the North-East) of those aged 80  years and older  had received the recommended number of doses of vaccine. 

NUTS: nomenclature of territorial units for statistics.

^a ^Based on NUTS1 areas for Italy [6].

To account for the time-lag between vaccination and the development of immunity, we assumed a delay of 2 weeks for each of the vaccine doses [7,8]. Thus, we defined as partially vaccinated those in the period between 14 days post-first dose and 13 days post-second dose; and as fully vaccinated those who had received the second dose or a single dose least 14 days earlier.

## Estimation of events averted by the vaccination programme

To measure the events adverted, we obtained data on all notified COVID-19 cases exploiting the case-based national COVID-19 integrated surveillance system [9], and data from vaccine coverage through the national vaccination portal of the Ministry of Health [10], both updated on 11 November 2021. We focused on data covering the population aged 12 years and older, for the period between 11 January (week 2) and 30 September (week 38) 2021. The weekly number of COVID-19 cases, hospitalisations, ICU admissions and deaths averted by the vaccination campaign was estimated using a method widely used in the study of the impact of the vaccination during the influenza season [11,12] and recently applied to calculate vaccine-prevented COVID-19 deaths [13]. Details can be found in the Supplementary Material 2. 

The weekly number of observed COVID-19 cases, hospitalisations, ICU admissions and deaths were summarised by date of diagnosis or sampling since we were interested in measuring the number of cases hospitalised, admitted to ICU and died and not when these events took place. We included in our analysis only hospitalisations, ICU admissions and deaths that occurred within 30 days of the COVID-19 diagnosis, which account for ca 96%, 97%, and 88% of the total numbers reported in the study period, respectively (Supplementary Figure S1). All analyses were stratified by age group (< 60 years, 60–69 years, 70–79 years, and 80 years and older), and geographical macro area. 

The results were further analysed by splitting the study period into three phases (January–March, April–June, July–September) characterised by different epidemiological situations and different levels of vaccination coverage (Table S1).

Details about VE estimation, methods and results used in this study can be found in the Supplementary Material 3. We also performed a sensitivity analysis varying the VE in an interval of +/ − 10 percentage points, considering as max upper limit 100%. The results of the sensitivity analyses are presented as ranges of the estimated averted events to indicate uncertainties.

All the analysis were performed using R (version 4.1.1) [14]. The list of the R packages used is available in the Supplementary Material.

## COVID-19 cases, hospitalisations, ICU admissions and deaths observed and averted 

A total of 445,193 (range: 331,059–616,054) cases, 79,152 (range: 53,209–148,756) hospitalisations, 9,839 (range: 6,434–16,276) ICU admissions and 22,067 (range: 13,571–48,026) deaths were estimated to have been averted by the vaccination campaign (Table), which account for 17% (range: 14%–23%), 32% (range: 24%–47%), 29% (range: 21%–41%) and 38% (range: 28%–57%) of the expected events (observed plus averted), respectively.

**Table:** Cumulative number of COVID-19 cases, hospitalisations, ICU admissions and deaths observed and averted by vaccination and observed and expected incidence rates, by geographical macro area and age group, Italy, week 2/2021−week 38/2021

Geographical macro area^a^	Age group (years)	COVID-19 cases						Hospitalisations						ICU admissions						Deaths					
		Observed	Averted		Observed incidence rate	Expected incidence rate		Observed	Averted		Observed incidence rate	Expected incidence rate		Observed	Averted		Observed incidence rate	Expected incidence rate		Observed	Averted		Observed incidence rate	Expected incidence rate		
		n	n	Range (+/-10% VE)^b^	Per 100,000	Per 100,000	Range (+/-10% VE)^b^	n	n	Range (+/-10% VE)^b^	Per 100,000	Per 100,000	Range (+/-10% VE)^b^	n	n	Range (+/-10% VE)^b^	Per 100,000	Per 100,000	Range (+/-10% VE)^b^	n	n	Range (+/-10% VE)^b^	Per 100,000	Per 100,000	Range (+/-10% VE)^b^	
**South and Islands**	< 60	516,578	69,467	53,618–89,093	4,183.7	4,746.2	4,617.9–4,905.2	14,980	4,674	3,669–5,636	121.3	159.2	151.0–167.0	1,437	376	296–420	11.6	14.7	14.0–15.0	1,040	153	120–185	8.4	9.7	9.4–9.9	
	60–69	79,060	14,309	10,554–19,794	3,051.5	3,603.7	3,458.8–3,815.5	9,150	3,303	2,309–4,625	353.2	480.6	442.3–531.7	1,694	658	449–821	65.4	90.8	82.7–97.1	1,983	519	361–714	76.5	96.6	90.5–104.1	
	70–79	50,634	10,264	7,367–14,802	2,622.1	3,153.6	3,003.6–3,388.6	9,783	3,699	2,450–6,374	506.6	698.2	633.5–836.7	1,835	711	453–1,084	95	131.8	118.5–151.2	3,646	1,067	686–1,686	188.8	244.1	224.3–276.1	
	≥ 80	33,433	14,557	10,478–21,227	2,433.5	3,493.0	3,196.1–3,978.5	8,635	5,622	3,938–8,727	628.5	1,037.7	915.1–1,263.7	640	442	309–661	46.6	78.8	69.1–94.7	5,322	2,718	1,896–3,962	387.4	585.2	525.4–675.8	
**Centre**	< 60	312,392	79,522	60,797–103,397	4,457.2	5,591.8	5,326.0–5,934.0	11,095	4,791	3,678–5,899	158.3	226.7	210.8–242.5	1,058	519	395–590	15.1	22.5	20.7–23.5	402	181	138–223	5.7	8.3	7.7–8.9	
	60–69	43,696	15,881	11,372–23,013	2,944.3	4,014.3	3,710.5–4,494.9	5,177	3,980	2,578–6,173	348.8	617.0	522.5–764.8	943	892	550–1,196	63.5	123.6	100.6–144.1	766	616	399–937	51.6	93.1	78.5–114.7	
	70–79	30,040	12,134	8,564–18,078	2,479.5	3,481.1	3,186.4–3,971.7	6,088	4,628	2,896–9,379	502.5	884.5	741.6–1,276.7	1,228	954	567–1,656	101.4	180.1	148.2–238.0	1,728	1,338	806–2,407	142.6	253.1	209.2–341.3	
	≥ 80	25,495	20,145	13,184–35,990	2,653.5	4,750.1	4,017.9–6,387.0	7,505	9,082	5,332–25,552	781.10	1,726.3	1,333.5–3,433.9	555	739	398–2,310	57.8	134.7	99.0–297.6	3,704	4,786	2,666–13,483	385.5	883.6	661.7–1,785.4	
**North-West**	< 60	404,442	74,552	57,078–96,666	4,189.3	4,961.5	4,780.5–5,190.5	20,276	5,140	3,969–6,294	210	263.3	251.1–275.2	2,386	511	394–578	24.7	30.0	28.8–30.7	327	156	120–192	3.4	5.0	4.6–5.4	
	60–69	63,895	13,641	9,952–19,116	3,144.4	3,815.7	3,634.2–4,085.2	10,441	3,444	2,368–4,897	513.8	683.3	630.4–754.8	2,398	744	497–936	118	154.6	142.5–164.1	929	413	284–576	45.7	66.0	59.7–74.1	
	70–79	47,812	10,162	7,242–14,775	2,834.3	3,436.7	3,263.6–3,710.1	12,585	3,842	2,522–6,699	746	973.8	895.5–1,143.1	2,772	730	461–1,125	164.3	207.6	191.7–231.0	2,578	1,029	656–1,638	152.8	213.8	191.7–249.9	
	≥ 80	37,192	18,608	12,552–31,063	2,836.7	4,256.0	3,794.1–5,206.0	13,505	8,551	5,326–18,641	1,030.1	1,682.3	1,436.3–2,451.9	1,945	566	340–1,278	148.4	191.5	174.3–245.8	5,945	4,256	2,556–8,836	453.4	778.1	648.4–1,127.4	
**North-East**	< 60	353,407	55,663	43,075–71,220	5,149.8	5,960.9	5,779.5–6,189.8	11,927	3,887	3,051–4,700	173.8	230.4	218.3–242.4	1,597	377	296–423	23.3	28.8	27.6–29.4	259	55	44–67	3.8	4.6	4.4–4.8	
	60–69	48,939	9,890	7,192–13,971	3,383.3	4,067.0	3,880.5–4,349.2	7,018	2,446	1,669–3,542	485.2	654.3	600.6–730.0	1,473	525	346–670	101.8	138.1	125.8–148.2	600	267	182–380	41.5	59.9	54.1–67.8	
	70–79	34,418	8,485	6,114–12,259	2,892.8	3,605.9	3,406.7–3,923.1	8,727	3,074	2,041–5,464	733.5	991.9	905.0–1,192.7	1,528	583	371–917	128.4	177.4	159.6–205.5	1,682	633	407–1,030	141.4	194.6	175.6–227.9	
	≥ 80	28,797	17,911	11,920–31,590	3,135.9	5,086.3	4,422.2–6,558.6	10,771	8,987	5,413–26,154	1,172.9	2,151.6	1,757.7–4,010.4	277	513	312–1,611	30.2	86.0	64.0–205.1	4,717	3,880	2,250–11,710	513.7	936.2	756.7–1,784.1	
**Italy**	< 60	1,586,819	279,205	214,568–360,376	4,423.4	5,201.7	5,022.2–5,428.7	58,278	18,493	14,367–22,529	162.5	214.0	202.5–225.3	6,478	1,783	1,381–2,011	18.1	23.0	21.9–23.7	2,028	545	422–667	5.7	7.2	6.8–7.5	
	60–69	235,590	53,721	39,070–75,894	3,119.	3,830.2	3,636.2–4,123.7	31,786	13,174	8,924–19,237	420.8	595.2	539–675.5	6,508	2,818	1,842–3,623	86.2	123.5	110.5–134.1	4,278	1,815	1,226–2,607	56.6	80.7	72.9–91.2	
	70–79	162,904	41,045	29,287–59,914	2,706.4	3,388.3	3,192.9–3,701.7	37,183	15,243	9,909–27,916	617.7	871.0	782.4–1,081.5	7,363	2,978	1,852–4,782	122.3	171.8	153.1–201.8	9,634	4,067	2,555–6,761	160.1	227.6	202.5–272.4	
	≥ 80	124,917	71,222	48,134–119,870	2,736.9	4,297.4	3,788–5,358.3	40,416	32,242	20,009–79,074	885.5	1,591.9	1,322.7–2,615.6	3,417	2,260	1,359–5,860	74.9	124.4	104.5–203.1	19,688	15,640	9,368–37,991	431.4	774.0	636–1,262.6	
**Total**		**2,110,230**	**445,193 **	**331,059**–**616,054**	**3,907.0**	**4,731.0 **	**4,520.0**–**5,048.0**	**167,663**	**79,152 **	**53,209**–**148,756**	**310.0**	**475.0 **	**409.0**–**586.0**	**23,766**	**9,839 **	**6,434**–**16,276**	**44.0**	**62.2 **	**55.9**–**74.1**	**35,628**	**22,067 **	**13,571**–**48,026**	**66.0**	**107.0 **	**91.1**–**155.0**	

COVID-19: coronavirus disease; ICU: intensive care unit: NUTS: nomenclature of territorial units for statistics; VE: vaccine effectiveness.

^a ^Based on NUTS1 areas for Italy [6].

^b^ Represent results of the sensitivity analysis, with +/- 10% vaccine effectiveness.

### Age-stratified hospitalisations, ICU admissions and deaths

Without vaccination, the expected hospitalisation rate would have been 214, 595, 871, 1,592 per 100,000 respectively for those aged under 60, 60–69, 70–79 and 80 years and older vs the observed rate of 163, 421, 618, 886 per 100,000 (ranges see Table). In terms of admissions to ICU, we observed a differences of 5 (range: 4−6), 37 (range: 24−48), 50 (range: 31−80) and 50 (range: 30−128) events per 100,000 between the expected and the observed cumulative rate among those under 60, 60–69, 70–79 years old and those aged 80 years and older, respectively. We estimated that 71% (range: 69−79) of the overall deaths were averted for those aged 80 years and older, and that 18% (range: 14-19), 8% (range: 5−9) and 2% (range: 1−3) were averted for those aged 70–79, 60–69 and under 60 years, respectively.

### COVID-19 cases, hospitalisations, ICU admissions and deaths by geographical macro area

**Figure 2:**
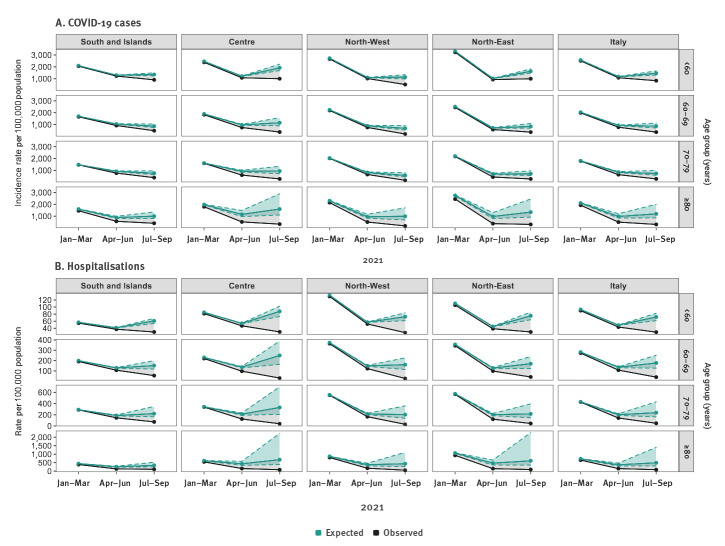
Cumulative and expected incidence rate (A) and cumulative and expected hospitalisation rate (B) with uncertainty ranges^a^, by period, age group and geographical macro area, Italy, week 2/2021−week 38/2021

COVID-19: coronavirus disease; ICU: intensive care unit.

^a^ Represent results of the sensitivity analysis, with +/- 10% vaccine effectiveness.

**Figure 3:**
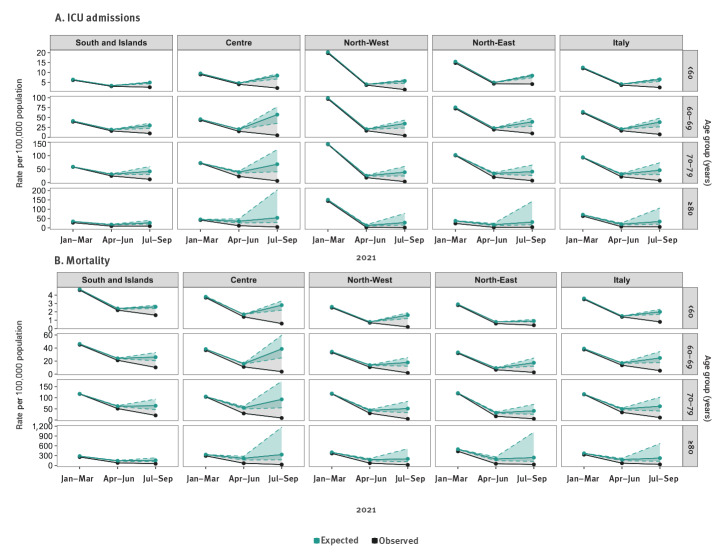
Cumulative and expected ICU admission rate (A) and cumulative and expected mortality rate (B) with uncertainty ranges^a^, by period, age group and geographical macro area, Italy, week 2/2021−week 38/2021

We observed large differences between observed and expected cumulative rates for the four studied outcomes by geographical macro area according to their vaccination coverage (Figures 2 and 3). Areas that achieved high vaccination coverage (around 90%) by the end of June in those aged 80 years and older (North-East, North-West and Centre) already had an estimated percentage of averted events for all outcomes together of over 50% in the period between April and June. 

ICU: intensive care unit.

^a^ Represent results of the sensitivity analysis, with +/- 10% vaccine effectiveness.

Without vaccination, between July and September, the overall expected mortality rate for those aged 80 years and older would have been 224 (range: 128−669) per 100,000 vs the observed rate of 32 per 100,000 during the same months (Figure 3). In the South and Islands we observed the lowest difference between the expected, 157 (range: 117−233) and the observed mortality rate, 52; whereas in the Centre we observed the largest difference, 332 (range: 170–1,170) vs 27. In the same period, for people aged 60−69 and 70–79 years in all the geographical areas, we estimated a percentage of averted hospitalisations and ICU admissions higher than 60%. Furthermore, for those aged under 60 years, the observed mortality rate and the observed hospital rate was less than half of the expected one by week 38 at the end of September, in all the geographical areas. 

Overall, we estimated that 74% (range: 72−77), 70% (range: 66−80), 75% (range: 71−82) and 62% (range: 55−78) of cases, hospitalisations, ICU admissions and deaths were, respectively, averted between July and September, given that the average full vaccination coverage at the end of September was higher than 60% in all age groups. Indeed in this period 48% (range: 40−57), 73% (range: 63−85), 78% (range: 68−86) and 83% (range: 73−93) of the expected cases, hospitalisations, ICU admissions and deaths were averted, respectively.

### Ethical statement

The dissemination of COVID-19 surveillance data was authorised by the Italian Presidency of the Council of Ministers on 27 February 2020 (Ordinance number 640).

## Discussion

The pace of the roll-out of COVID-19 mass vaccination varied by age group and across geographical macro areas in Italy, particularly in people aged 80 years and older, and influenced the magnitude of prevented infections, hospitalisations, ICU admissions and deaths. The South-Islands experienced less averted events than other macro areas mainly because of a slower vaccination uptake in those at higher risk and the high incidence of COVID-19 cases observed during the tourist season (July−August). Rates of expected and observed events for all four outcomes started to diverge in the period between January and March in those aged 80 years older; and between April and June in the other age groups. Our model estimations show that without vaccination, peaks in hospitalisations, ICU admissions and deaths higher than those observed would have been detected for people aged 80 years and older starting from April and for other age groups from July to September. Overall, the largest proportions of hospitalisations and deaths prevented by the vaccination was observed in the oldest age group (41%; range 38−53 and 71%; range 69−79, respectively), whereas the largest number of averted ICU admissions has been observed in those aged between 60 and 79 years (59%; range: 52−57).

Our results are consistent with the current literature that demonstrates a positive impact of COVID-19 vaccination in preventing infections [4] and severe disease [13,15], with a larger reduction in the COVID-19 burden in older adults [16,17]. Furthermore, previous studies have estimated the number of deaths averted as a result of the vaccination roll-out [13,15,17,18]. However, to the best of our knowledge, this is the first study that, exploiting a standard approach, estimates the impact of the COVID-19 vaccinations in terms of prevented events in Italy for all the age groups eligible for vaccination and which analyses geographical differences.

The analysis has several limitations. The method used assumes that vaccination impact is only driven by its direct effects and does not take into account its potential indirect effects such as impact on the overall transmissibility and/or relaxation of restriction measures. The proposed calculation may therefore have underestimated the number of avoided events. Moreover, since our approach is not based on a dynamic-transmission model, it is not able to predict future behavioural changes of the population in the counterfactual situation of no-vaccination having been available in 2021, as Italy may have implemented multiple restriction measures over 2021 had the vaccines not been available. Although we performed a sensitivity analysis to determine how different values of VE affect the estimates, we did not take into account other factors that have been found to influence VE, such as the vaccine type [19]. Finally, concurrent with the start of the vaccination roll-out, various non-pharmaceutical interventions were introduced to control the spread of the virus. Both the measures and the vaccination uptake are likely to have had an impact on the incidence of COVID-19 cases, hospitalisations, ICU admissions and deaths.

### Conclusion

Our findings show a positive impact of the COVID-19 vaccination programme in Italy, and suggest that the rapid vaccination of high-risk groups has prevented a considerable number of severe COVID-19 outcomes. Averted hospitalisations and ICU admissions ranged between 53,209 and 148,756 and 6,434 and 16,276, respectively, and for deaths averted the range was 13,571–48,026. Geographical areas that achieved high vaccination rates faster were able to prevent a larger number of hospitalisations, ICU admissions and deaths over the summer months.

## Supplementary Data

2706000 Supplementary Material
