# Supplementary material for: Estimating averted COVID-19 cases, hospitalisations, intensive care unit admissions and deaths by COVID-19 vaccination, Italy, January−September 2021
Source: Euro Surveill. 2021 Nov 25;26(47):2101001. doi: 10.2807/1560-7917.ES.2021.26.47.2101001 (PMC8619872; doi:10.2807/1560-7917.ES.2021.26.47.2101001)
Supplement: Supplementary Material [file 21-01001_SACCO_Supplementary_Material.pdf]

Supplementary materials to the article:

Estimating COVID-19 events averted by the vaccination campaign in Italy: an evaluation in the period January- September 2021

This supplementary material is hosted by *Eurosurveillance* as supporting information alongside the article 'Estimating averted COVID-19 cases, hospitalisations, intensive care unit admissions and deaths by COVID-19 vaccination, Italy, January−September 2021', on behalf of the authors, who remain responsible for the accuracy and appropriateness of the content. The same standards for ethics, copyright, attributions and permissions as for the article apply. Supplements are not edited by *Eurosurveillance* and the journal is not responsible for the maintenance of any links or email addresses provided therein.

Supplementary Material 1

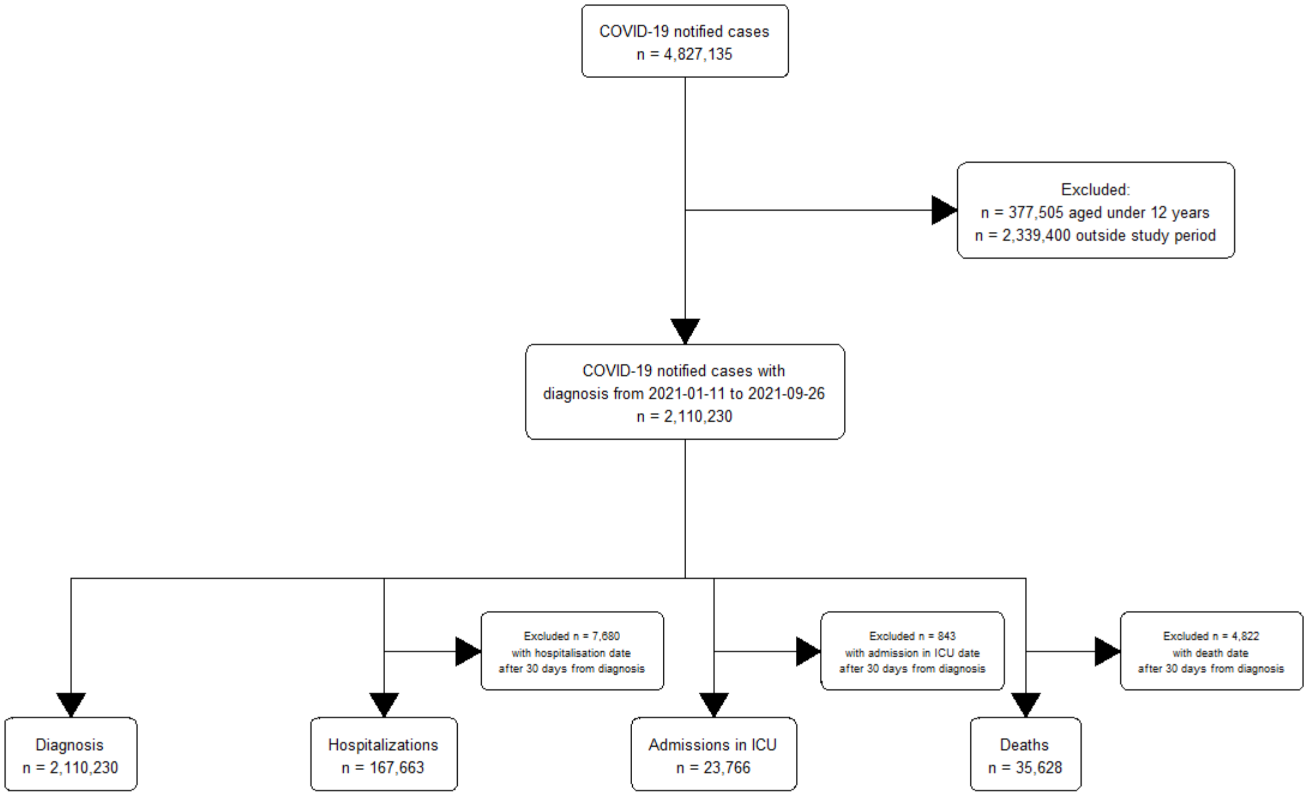

Figure S1. Flow chart of the study population from the COVID-19 Integrated Surveillance System for assessment of the study events (Data extracted on 11 November 2021).

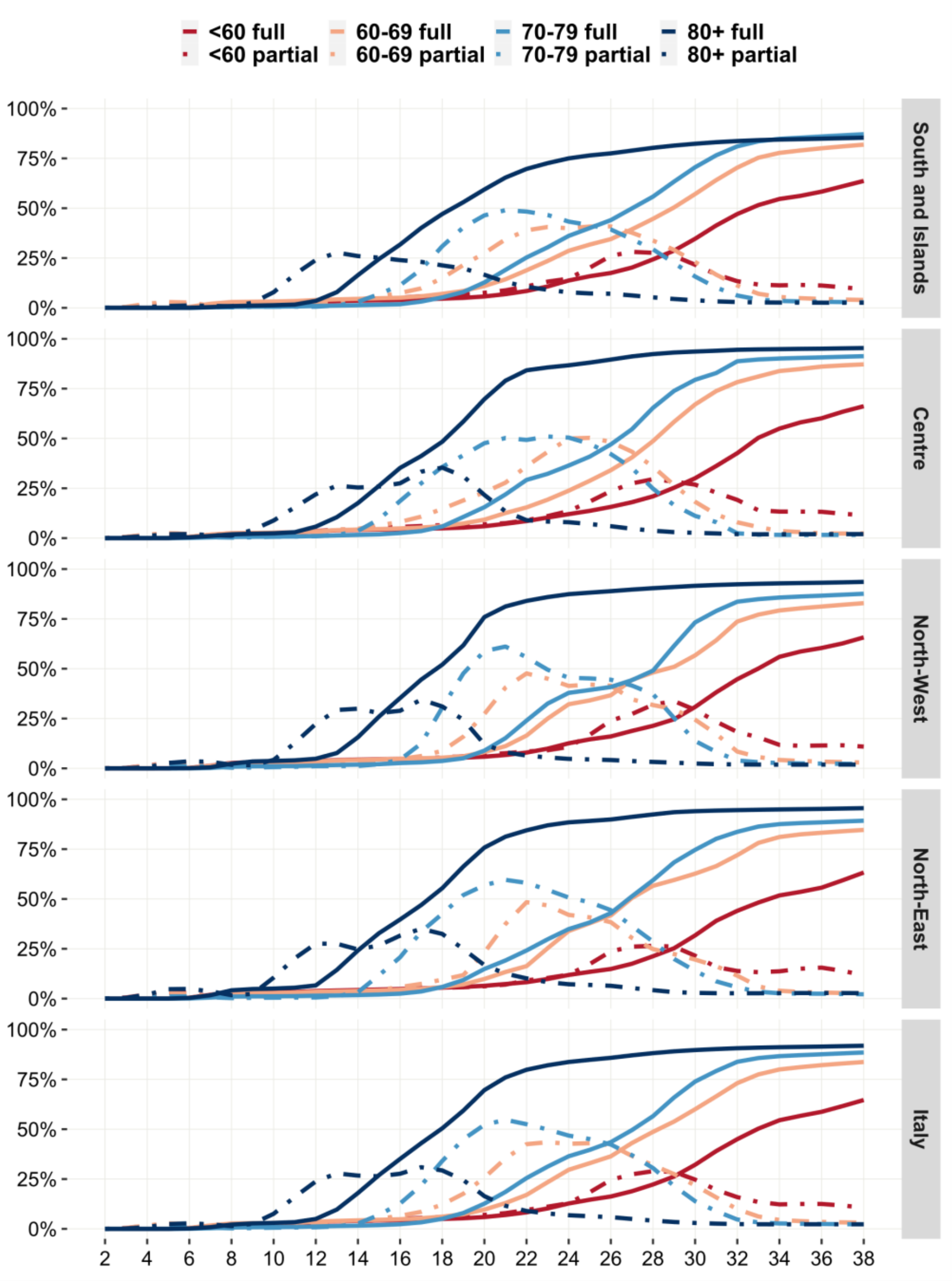

Figure S2. Cumulative weekly full and partial vaccination coverage by age group (<60, 60-69, 70-79, 80+) and geographical area (Italy, North-West, North-East, Centre, South and Islands) between week 2/2021 and week 38/2021.

Table S1. Characterization of study periods (Jan-Mar; Apr-Jun; Jul-Sep) by full vaccination coverage and incidence rate by age group.

Period	Age group	Full vaccination coverage (%)	Cumulative incidence rate per 100,000	SARS-CoV-2 variant
**Jan - Mar**	<60	3.8	2,510	Alpha
	60-69	4.0	1,984	
	70-79	1.4	1,788	
	80+	9.7	1,929	
**Apr - Jun**	<60	16.2	1,080.8	Alpha
	60-69	36.4	774.9	
	70-79	43.5	638.0	
	80+	85.8	502.2	
**Jul - Sep**	<60	64.6	832.5	Delta
	60-69	83.7	359.8	
	70-79	88.5	280.2	
	80+	91.9	306.0	

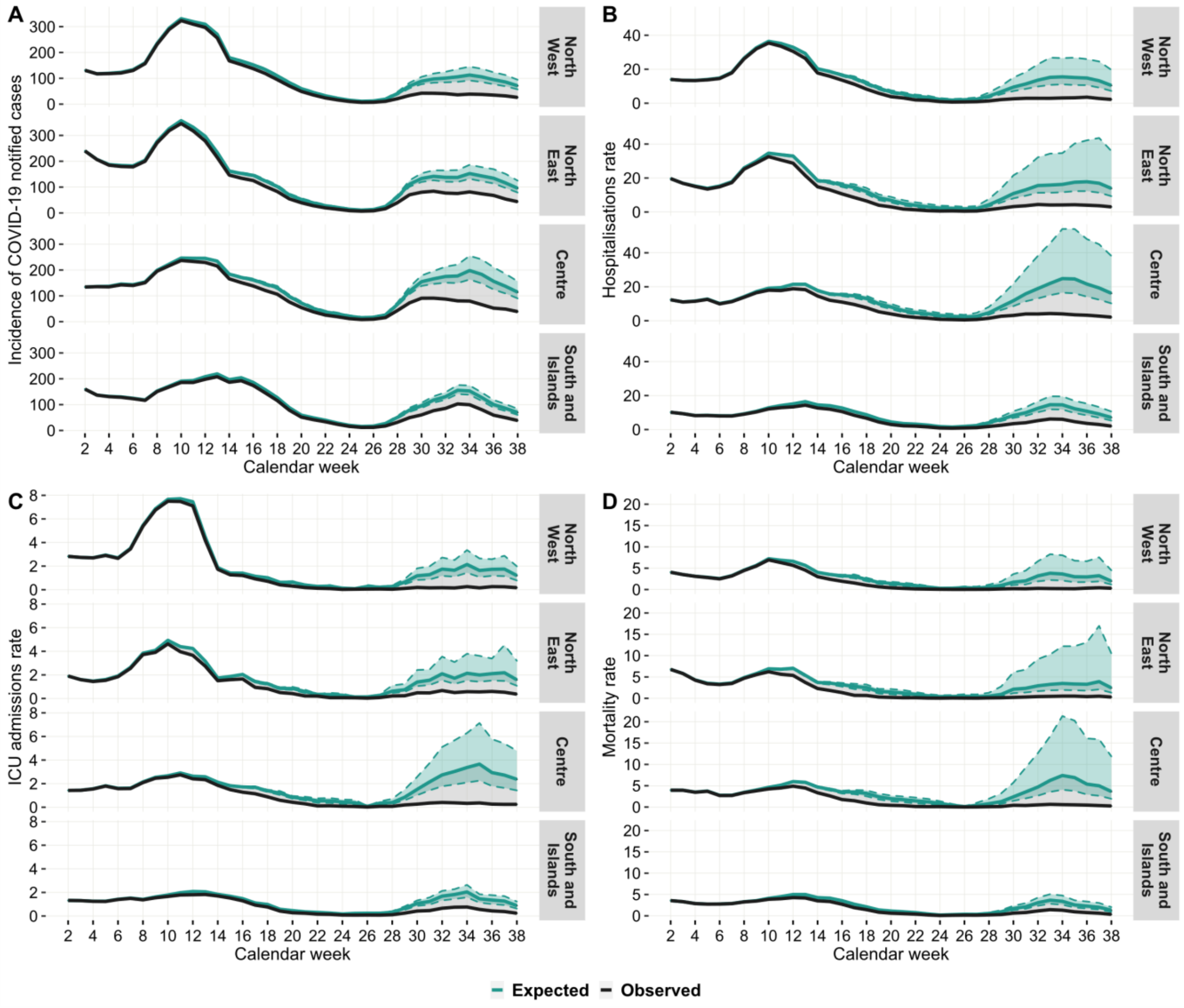

Figure S3. Weekly observed and expected (+/- 10% Vaccine Effectiveness) rate per 100.000 people of COVID-19 notified cases (A), hospitalisations (B), ICU admissions (C), deaths (D) in Italy between week 2/2021 to week 38/2021 by geographical area.

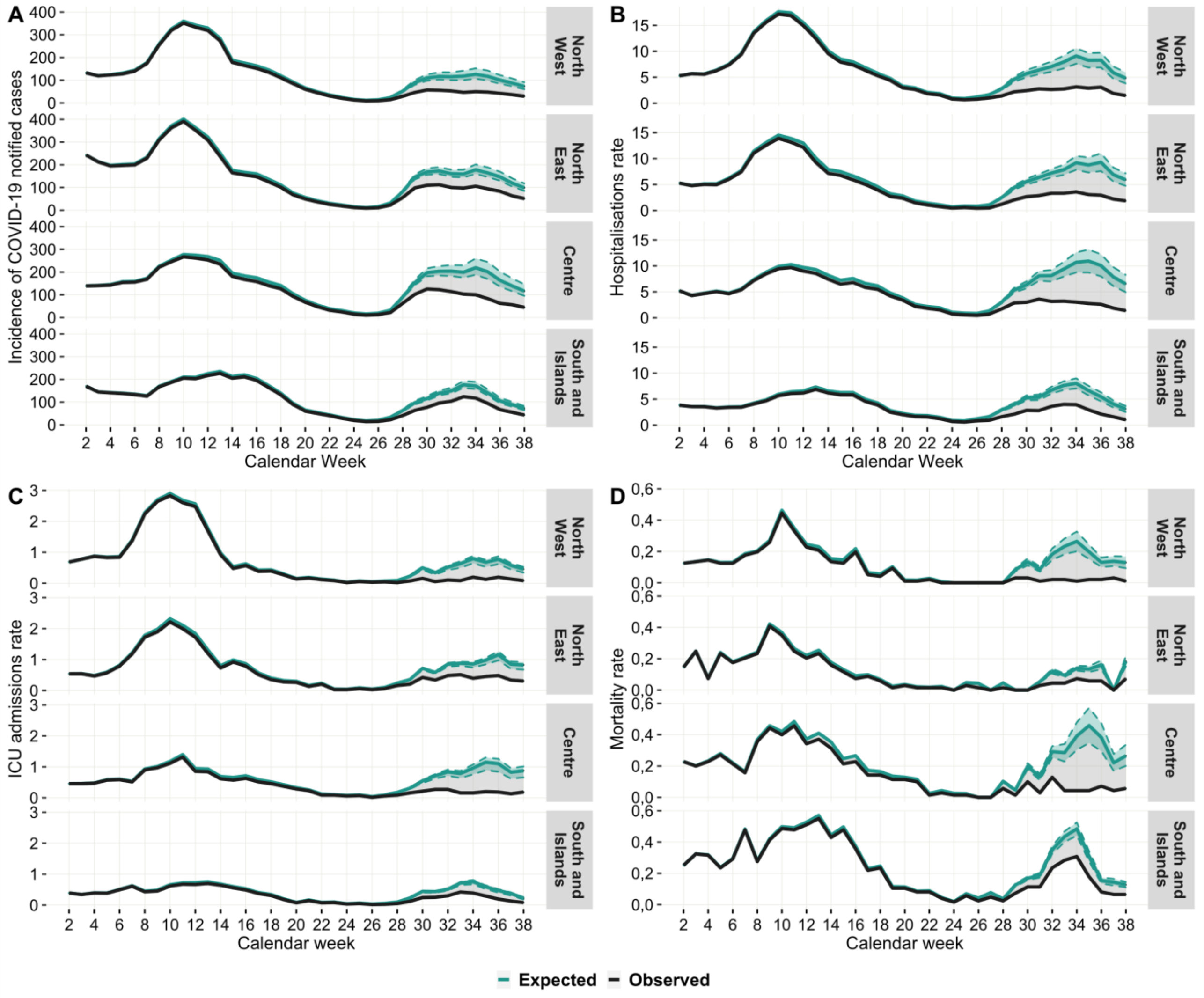

Age group: <60

Figure S4. Weekly observed and expected (+/- 10% Vaccine Effectiveness) rate per 100.000 people of COVID-19 notified cases (A), hospitalisations (B), ICU admissions (C), deaths (D) between week 2/2021 to week 38/2021 by geographical area for those aged under 60 years.

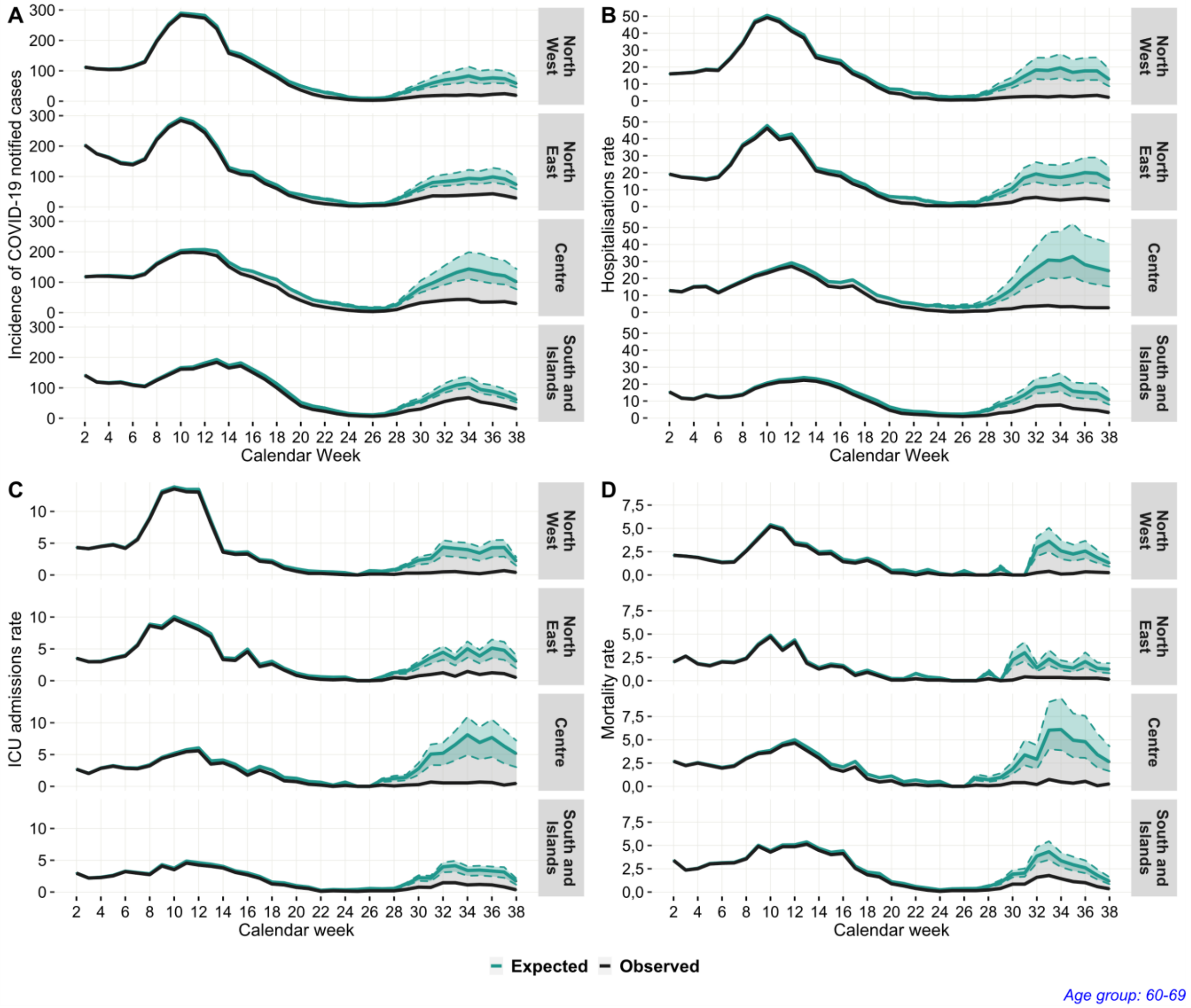

Figure S5. Weekly observed and expected (+/- 10% Vaccine Effectiveness) rate per 100.000 people of COVID-19 notified cases (A), hospitalisations (B), ICU admissions (C), deaths (D) between week 2/2021 to week 38/2021 by geographical area for those aged between 60-69 years.

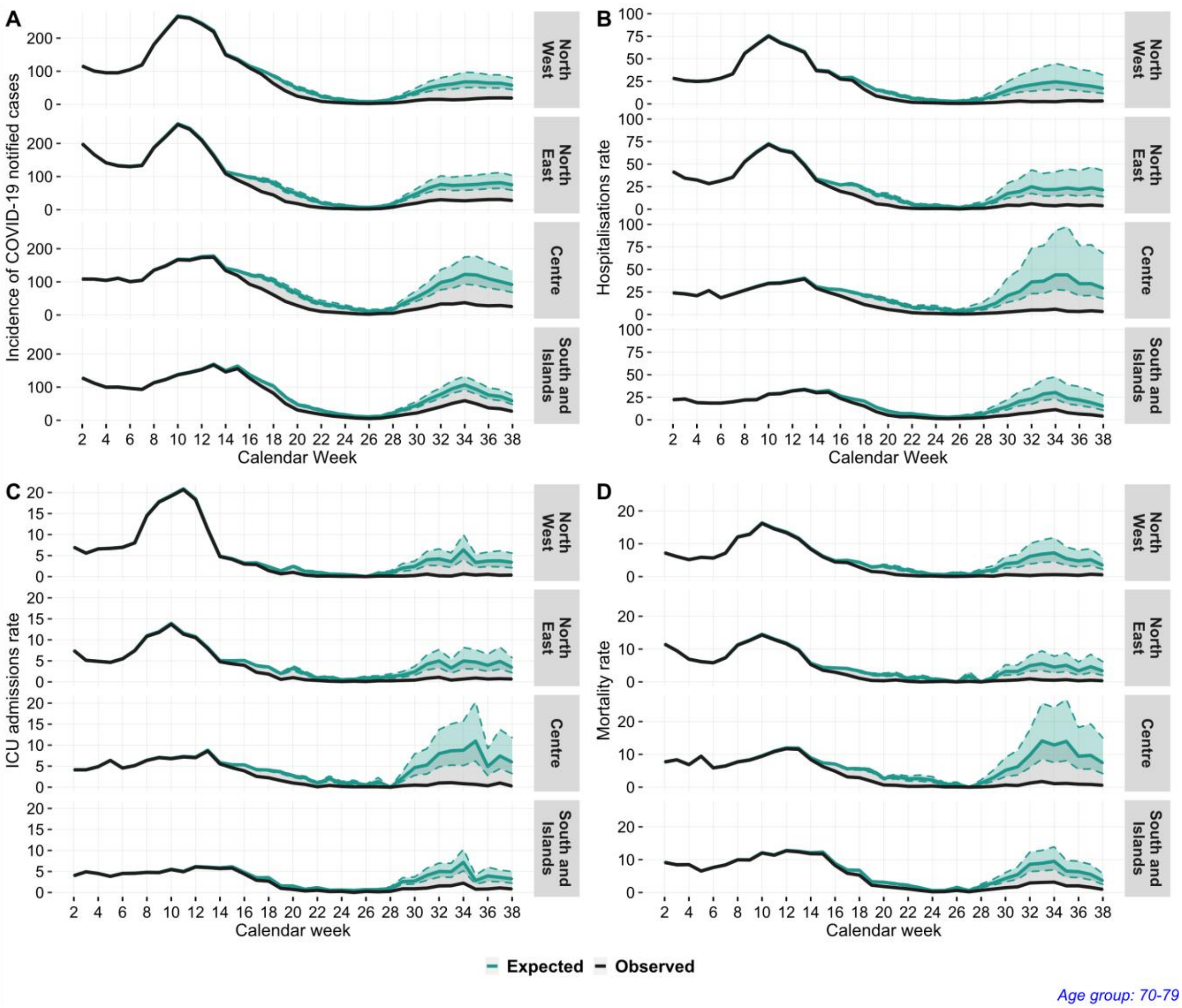

Figure S6. Weekly observed and expected (+/- 10% Vaccine Effectiveness) rate per 100.000 people of COVID-19 notified cases (A), hospitalisations (B), ICU admissions (C), deaths (D) between week 2/2021 to week 38/2021 by geographical area for those aged between 70-79 years.

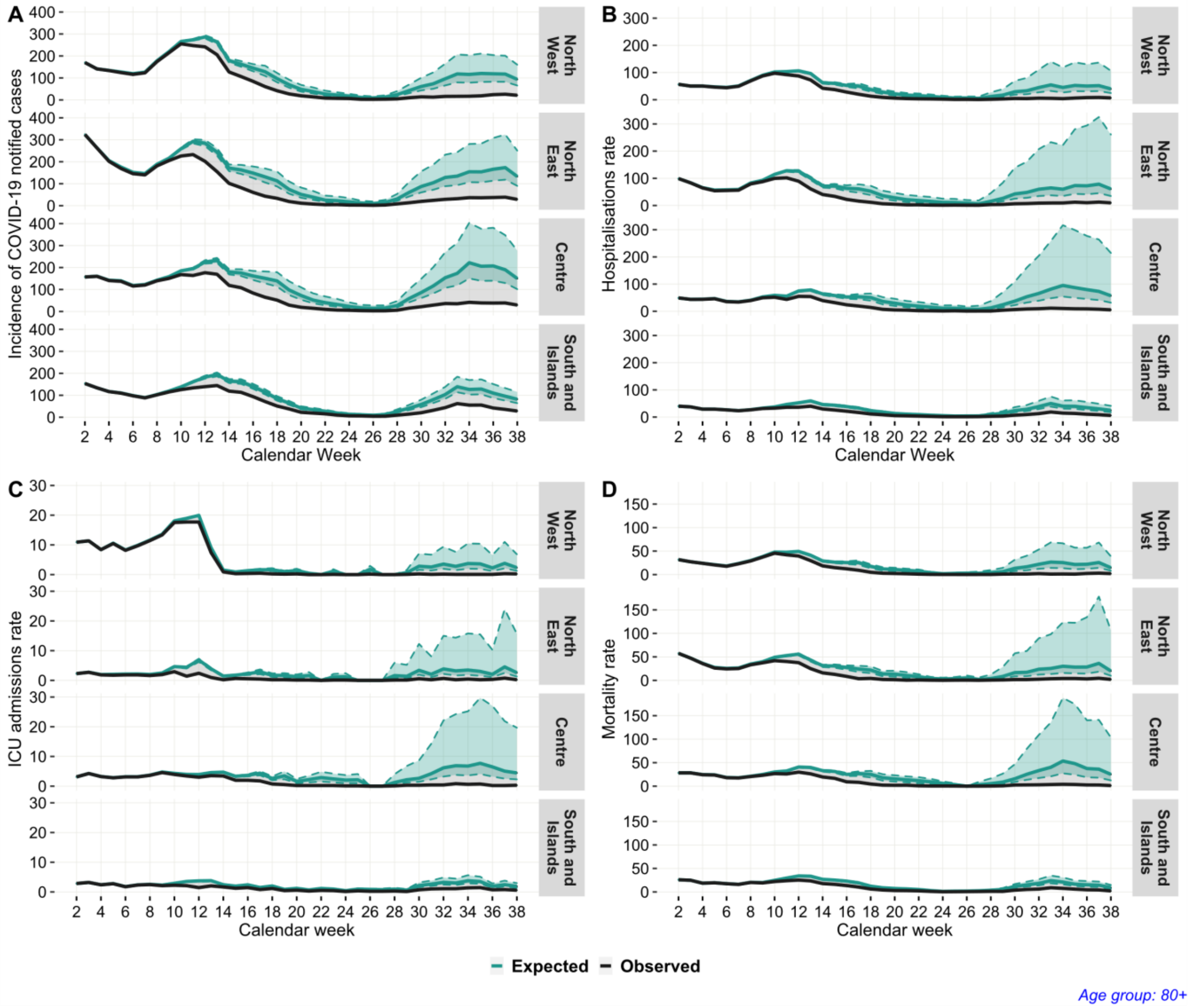

Figure S7. Weekly observed and expected (+/- 10% Vaccine Effectiveness) rate per 100.000 people of COVID-19 notified cases (A), hospitalisations (B), ICU admissions (C), deaths (D) between week 2/2021 to week 38/2021 by geographical area for those aged 80 years and over.

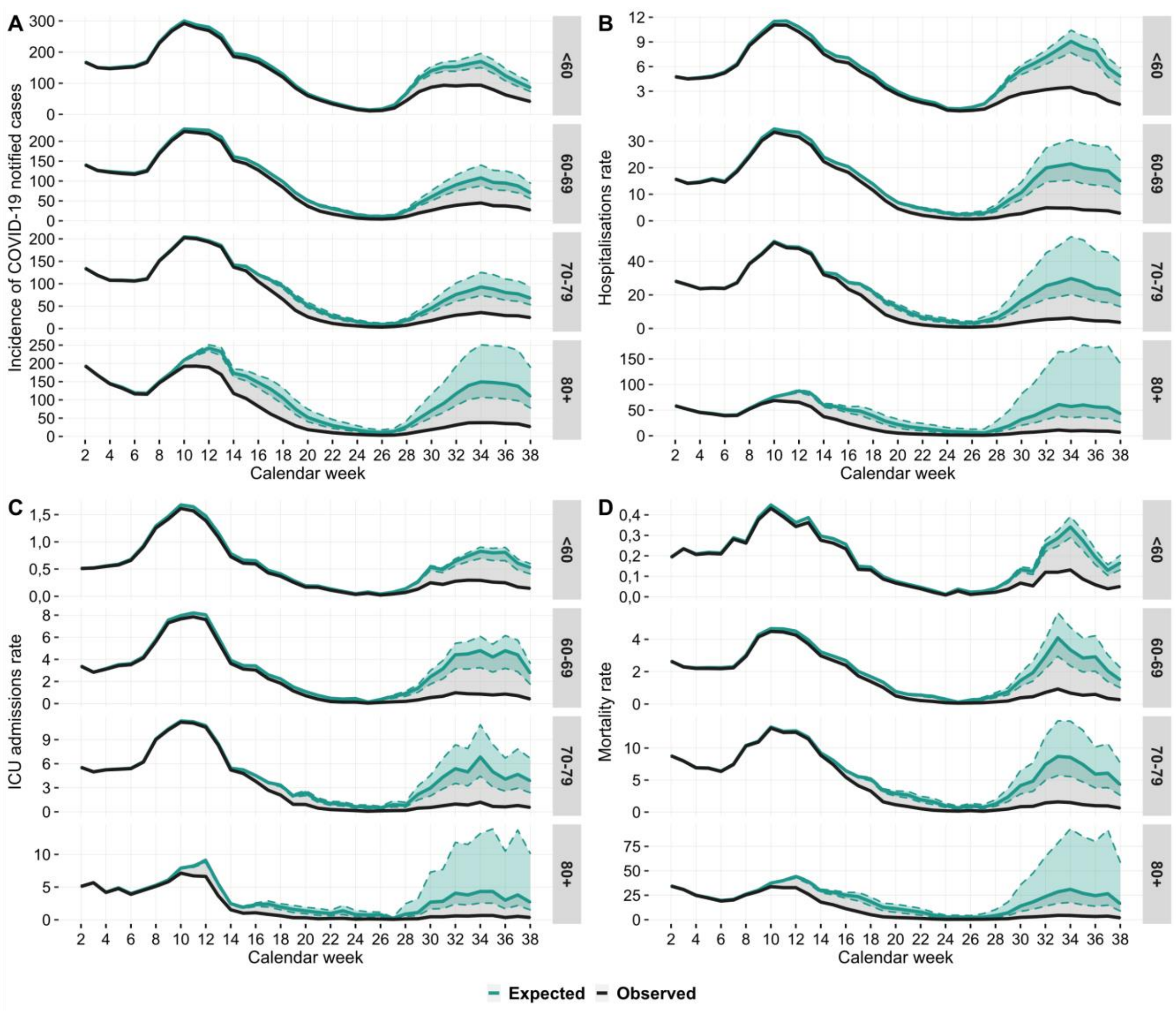

Figure S8. Weekly observed and expected (+/- 10% Vaccine Effectiveness) rate per 100.000 people of COVID-19 notified cases (A), hospitalisations (B), ICU admissions (C), deaths (D) in Italy between week 2/2021 to week 38/2021 by age group.

Supplementary Material 2: Estimation of averted events

The weekly number of COVID-19 infections, hospitalisations, ICU admissions and deaths averted by the vaccination campaign in Italy was estimated using a method widely used in the study of the impact of the vaccination during the flu season and recently exploited by Public Health England to calculate vaccine-prevented SARS-CoV-2 deaths. This approach is based on the idea that the weekly impact of the vaccination on the studied event (COVID-19 notified cases, hospitalisations, ICU admissions and deaths) can be estimated exploiting VE against the event of interest, the weekly vaccinations coverage and the weekly number of observed events as follows:

$$NAV_k = \frac{N * VE_k VC_k}{1 - \sum_{k=1}^{2} VE_k VC_k}$$

where NAV_k is the weekly number of averted events by the vaccination dose k (with $k = 1,2$), N is the weekly rolling average number of events observed over three weeks, VE_k is the vaccine effectiveness at the k vaccination dose and VC_k is the weekly vaccine coverage after the dose k.

Supplementary Material 3: Estimation of the vaccine effectiveness

The vaccine effectiveness is computed exploiting the same approach proposed in the weekly report of the Italian National Institute of Health (available on the following link: https://www.epicentro.iss.it/coronavirus/sars-cov-2-sorveglianza-dati). Data are obtained by the weekly linking of COVID-19 data on SARS-CoV-2 infections notified to the National COVID-19 Integrated Surveillance System with vaccinated persons listed in the National Vaccination Registry of the Ministry of Health. We defined as partially vaccinated who received only the first dose of a two-dose vaccine (Comirnaty, Spikevax and Vaxzevria vaccines) after 14 days, or who received the second dose from ≤14 days and as fully vaccinated who received the second dose (Comirnaty, Moderna and Vaxzevria) or single dose (COVID-19 Vaccine Janssen) after at least 14 days. The study population includes all the COVID-19 notified cases from 5th April, 2021. According to the report on the SARS-CoV-2 variants of the Italian National Health Institute (available at the following link: https://www.epicentro.iss.it/coronavirus/pdf/sars-cov-2-monitoraggio-varianti-rapporti-periodici-23-luglio-2021.pdf), the delta variant resulted dominant at the beginning of July. Thus, VE was estimated separately for each epidemic phase (Alpha-phase, from 5th April 2021 to 4th July 2021, during which the circulation of the Alpha variant of the SARS-CoV-2 virus was dominant; and Delta-phase, from 5th July to 26th September 2021, during which the Delta variant was dominant). We estimated a Negative Binomial Generalized Linear Mixed Model (GLMM) assuming the daily number of events as dependent variable, vaccination status (unvaccinated, incompletely vaccinated, completed vaccinated) as independent variable, including the region of vaccination as random effect, with the offset being the log of the daily number of the persons exposed by vaccination status, region of vaccination. For model of the VE of those aged under 60 years, we adjusted for ten years age groups. The estimations of the vaccine effectiveness reported in the Table S2 are consistent with results in literature. Recent studies conducted in Scotland [1] and England [2] reported an effectiveness of complete vaccination against SARS-CoV-2 infection and severe COVID-19 of 79% (75% to 82%) and 96% (86% to 99%), respectively, during the Delta variant epidemic surge. Regarding the effectiveness of the partially vaccinated, we observed wider confidence interval with respect to the fully vaccinated estimates. Although the VE estimates for the partially vaccinated during the Delta variant epidemic phase could be affected by confounding factors that are not been included in the model, this did not affect the computation of the averted events since a really low percentage of the population was partially vaccinated during this period.

Table S2. Vaccine effectiveness (with +/- 10% uncertainty range used for the sensitivity analysis) by outcome, age group and vaccination status and epidemic phase.

Outcome	Age group	Alfa		Delta	
		Partially vaccinated vs unvaccinated	Fully vaccinated vs unvaccinated	Partially vaccinated vs unvaccinated	Fully vaccinated vs unvaccinated
Notified cases	<60	54.9 [44.9 - 64.9]	79.1 [69.1 - 89.1]	59.2 [49.2 - 69.2]	76.1 [66.1 - 86.1]
	60-69	63.2 [53.2 - 73.2]	85.9 [75.9 - 95.9]	61.6 [51.6 - 71.6]	75.3 [65.3 - 85.3]
	70-79	59.4 [49.4 - 69.4]	87.6 [77.6 - 97.6]	62.8 [52.8 - 72.8]	73.6 [63.6 - 83.6]
	80+	54.6 [44.6 - 64.6]	84.5 [74.5 - 94.5]	74.9 [64.9 - 84.9]	80.5 [70.5 - 90.5]
Hospitalizations	<60	88.5 [78.5 - 98.5]	93.2 [83.2 - 100]	89.0 [79.0 – 99.0]	94.6 [84.6 - 100]
	60-69	88.1 [78.1 - 98.1]	96.1 [86.1 - 100]	87.0 [77.0 – 97.0]	93.3 [83.3 - 100]
	70-79	81.4 [71.4 - 91.4]	94.3 [84.3 - 100]	84.0 [74.0 – 94.0]	90.5 [80.5 - 100]
	80+	69.3 [59.3 - 79.3]	94.2 [84.2 - 100]	80.5 [70.5 - 90.5]	89.0 [79.0 – 99.0]
ICU admissions	<60	92.5 [82.5 - 100]	98.4 [88.4 - 100]	94.1 [84.1 - 100]	96.6 [86.6 - 100]
	60-69	93.0 [83.0 - 100]	98.2 [88.2 - 100]	93.4 [83.4 - 100]	96.0 [86.0 - 100]
	70-79	88.9 [78.9 - 98.9]	97.9 [87.9 - 100]	92.4 [82.4 - 100]	93.4 [83.4 - 100]
	80+	76.8 [66.8 - 86.8]	96.0 [86 - 100]	86.6 [76.6 - 96.6]	91.2 [81.2 - 100]
Deaths	<60	85.2 [75.2 - 95.2]	92.5 [82.5 - 100]	89.3 [79.3 - 99.3]	94.6 [84.6 - 100]
	60-69	88.9 [78.9 - 98.9]	97.7 [87.7 - 100]	91.7 [81.7 - 100]	93.3 [83.3 - 100]
	70-79	88.1 [78.1 - 98.1]	95.9 [85.9 - 100]	87.9 [77.9 - 97.9]	93.1 [83.1 - 100]
	80+	75.6 [65.6 - 85.6]	96.4 [86.4 - 100]	83.1 [73.1 - 93.1]	92.0 [82.0 - 100]

References

1. Sheick A, McMenamin J, Taylor B, Robertson C. SARS-CoV-2 Delta VOC in Scotland: demographics, risk of hospital admission, and vaccine effectiveness. Lancet. 2021. Jun 26;397(10293):2461-2462
2. Stowe J, Andrews N, Gower C, et al. Effectiveness of COVID-19 vaccines against hospital admission with the Delta (B.1.617.2) variant. khub. 2021. https://khub.net/web/phe-national/public-library/-/document_library/v2WsRK3ZlEig/view/479607266

Supplementary Material 4: References of the R packages used

Bates D, Mächler M, Bolker B, Walker S (2015). "Fitting Linear Mixed-Effects Models Using lme4." *Journal of Statistical Software*, **67**(1), 1–48. doi: 10.18637/jss.v067.i01.

Brooks ME, Kristensen K, van Benthem KJ, Magnusson A, Berg CW, Nielsen A, Skaug HJ, Maechler M, Bolker BM (2017). "glmmTMB Balances Speed and Flexibility Among Packages for Zero-inflated Generalized Linear Mixed Modeling." *The R Journal*, **9**(2), 378–400. https://journal.r-project.org/archive/2017/RJ-2017-066/index.html.
